# The effect modification of PM_2.5_ and ozone on the short-term associations between temperature and mortality across the urban areas of Japan

**DOI:** 10.1265/ehpm.24-00108

**Published:** 2024-10-26

**Authors:** Muhammad Abdul Basit Ahmad Tajudin, Ryusei Kubo, Chris Fook Sheng Ng, Masahiro Hashizume, Xerxes Seposo, Yoonhee Kim, Hironori Nishikawa, Hirohisa Takano, Kayo Ueda

**Affiliations:** 1 Department of Hygiene, Graduate School of Medicine, Hokkaido University, Hokkaido, Japan; 2 Department of Environmental Engineering, Graduate School of Engineering, Kyoto University, Kyoto, Japan; 3 Department of Global Health Policy, School of International Health, Graduate School of Medicine, The University of Tokyo, Tokyo, Japan; 4 School of Tropical Medicine and Global Health, Nagasaki University, Nagasaki, Japan; 5 Ateneo Center for Research and Innovation, Ateneo School of Medicine and Public Health, Ateneo de Manila University, Pasig, Philippines; 6 Department of Global Environmental Health, Graduate School of Medicine, The University of Tokyo, Tokyo, Japan; 7 Graduate School of Global Environmental Studies, Kyoto University, Kyoto, Japan

**Keywords:** Temperature, Mortality, Air pollution, PM_2.5_, Ozone, Effect modification

## Abstract

**Background:**

The acute effects of temperature and air pollution on mortality are well-known environmental factors that have been receiving more recognition lately. However, the health effects resulting from the interaction of air pollution and temperature remain uncertain, particularly in cities with low levels of pollution. This study aims to examine the modification effects of particulate matter with a diameter of 2.5 µm or less (PM_2.5_) and ozone (O_3_) on the association between temperature and mortality.

**Methods:**

We collected the daily number of all-cause, cardiovascular, and respiratory mortality from 20 major cities in Japan from 2012–2018. We obtained meteorological data from the Japan Meteorological Agency and air pollution data from the National Institute for Environmental Studies. We conducted analyses using a quasi-Poisson regression model with a distributed lag non-linear model for temperature in each city and subsequently performed a random-effects meta-analysis to derive average estimates.

**Results:**

We found that high levels of O_3_ might positively modify the mortality risk of heat exposure, especially for cardiovascular diseases. Subgroups such as the elderly and females were susceptible. We did not observe consistent evidence of effect modification by PM_2.5_, including effect modification on cold by both pollutants.

**Conclusion:**

PM_2.5_ and O_3_ may positively modify the short-term association between heat and mortality in the urban areas of Japan. These results highlight the need for public health policies and interventions to address the collective impacts of both temperature and air pollution.

**Supplementary information:**

The online version contains supplementary material available at https://doi.org/10.1265/ehpm.24-00108.

## Graphical Abstract

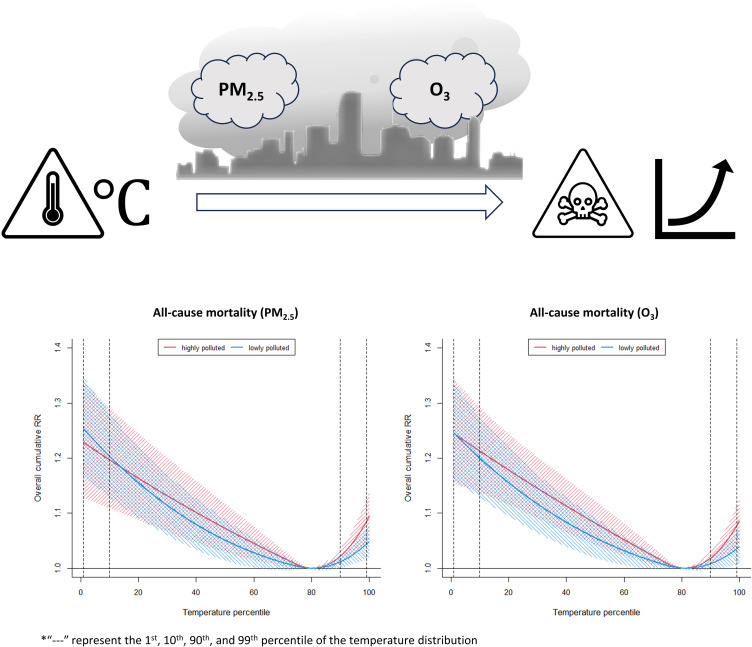

## Background

Climate change and air pollution are two main environmental challenges that affect human health. The increasing incidence and intensity of extreme temperature events, for example, heat waves and cold spells [1], contribute to excess mortality in many regions of the world [2–8]. Simultaneously, exposure to fine particulate matter (PM_2.5_) and ozone (O_3_), the principal constituents of urban air pollution, has also been linked to increased mortality and morbidity across various diseases [9–12]. However, the combined impacts of temperature and air pollution on mortality are not well understood, especially in urban areas where both factors may exhibit substantial spatial and temporal variations.

Temperature and air pollutants are often examined together in epidemiological studies, where one is controlled for the other as a confounding factor [13–15]. However, literature that focuses on the effect modification by air pollutants is relatively sparse in number and geographical scope [16–22], with fewer yet exploring the possible modification effects of PM_2.5_ and O_3_ [20, 22, 23]. Moreover, comparing these modification effects across studies proves challenging because of the variations in the country's climate, air pollution levels, as well as population characteristics.

Over the years, the yearly mean temperature in Japan has risen by 1.24 °C per century between 1898 and 2019. Days with very high temperatures (≥30 °C or ≥35 °C) have also increased significantly [24]. The annual mean temperature in Japan is projected to continue rising in the future, leading to an increase in hot days and associated health impacts, including heatstroke or cold spells, and additional mortality related to heat and cold [24, 25]. The PM_2.5_ concentration in Japan has been decreasing over the years; however, the trend of O_3_ seems to be increasing [26]. Although a previous Multi-Country Multi-City (MCC) Collaborative Research Network study has estimated the effect modification of PM_2.5_ and O_3_ on temperature, they focused only on the effect modification during high temperature [20]. Coupled with the rapidly urbanized cities, Japan provides a suitable study setting to look at the modifying effect of PM_2.5_ and O_3_ on both low and high-temperature-mortality associations. Addressing this uncertainty is crucial for shaping policy decisions aiming to improve air quality amid ongoing climate change. Therefore, we aim to investigate the potential effect modification of PM_2.5_ and O_3_ on the temperature and mortality association in urban centers of Japan. In addition, the consideration of variations in sex, age, and cause of death could further elucidate the complex interplay between air pollution on temperature [17, 27, 28].

## Methods

### Data collection

**Fig. 1:**
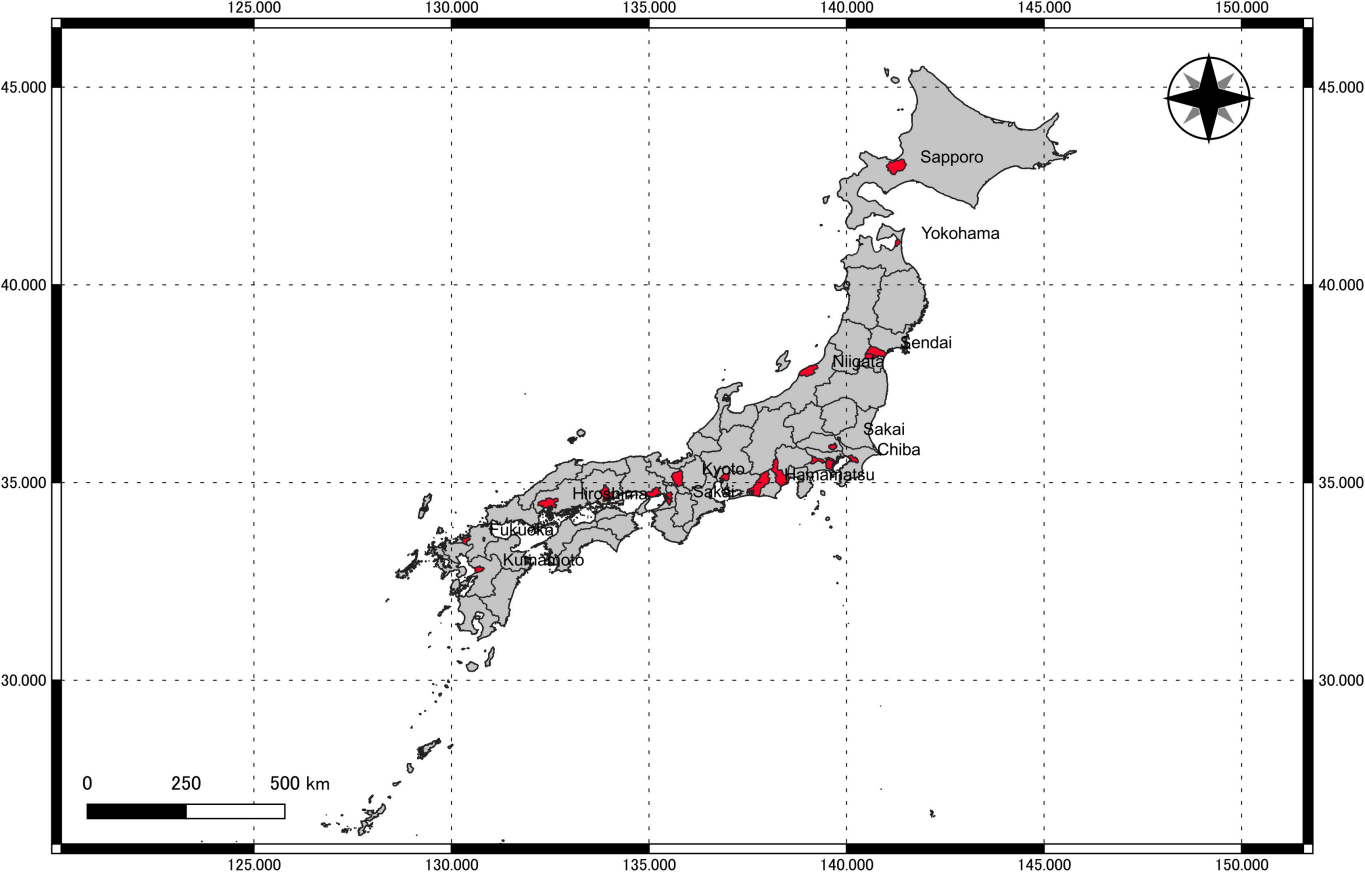
Study locations.

We collected daily mortality data from January 1^st^, 2012, to December 31^st^, 2018, encompassing 20 government ordinance-designated cities in Japan consisting of the date of death, cause of death (all causes, cardiovascular (International Classification of Diseases 10th revision (ICD-10): I00–I99), and respiratory causes (ICD-10: J00–J99)), age, and sex from Health, Labor, and Welfare. These cities are presented in Fig. 1. We also collected meteorological and air pollution data from the Japan Meteorological Agency and the National Institute for Environmental Studies (NIES), respectively. We identified monitoring stations in each city and extracted the daily mean temperatures, relative humidity, PM_2.5_ averages, and maximum 8-hour moving average oxidants. The term “photochemical oxidant” has previously been used in the context of air quality standards in Japan. However, in recent years, there has been a transition of methodology at the monitoring stations from measuring oxidants using the absorption photometry method to measuring ozone using the UV absorption method [29]. Therefore, this paper consistently employs the term ozone (O_3_) for effect modification analysis. We averaged data from multiple stations within the same city. The PM_2.5_ measurement period was shorter for some cities. We imputed missing air pollutant data, which accounted for less than 1%, using centered 3-day moving averages.

### Statistical analysis

We conducted a two-stage analysis to estimate the temperature-mortality association and its modification by air pollution in each city, followed by a random-effects meta-analysis to pool the results across cities.

In the first stage, we utilized a quasi-Poisson regression model with a distributed lag non-linear model (DLNM) to capture the non-linear and delayed effects of temperature in each city [30]. This approach allows us to assess both the immediate effects of high temperatures and the delayed effects of low temperatures that can last for several weeks [15, 31, 32]. We used a quadratic B-spline with one knot at the 75^th^ percentile for temperature, based on previous studies that suggested a J-shaped temperature-mortality relationship, with the minimum mortality temperature around the 70^th^–80^th^ percentile [33]. We used a 21-day lag period with 3 equally spaced internal knots at the log scale for the delayed effects.

To examine the effect modification by air pollutants, we introduced an interaction term between temperature and a binary variable indicating high or low levels of air pollutants. We defined high level as above the 75^th^ percentile of daily concentrations across all 20 cities, and low level as below or equal to this threshold. This way, we could compare the temperature-mortality association under different air pollution conditions.

We controlled seasonality and long-term trends, day of the week, and air pollutant levels. The model is as follows:

$$Y_{t}∼\text{Poisson}(\mu_{t})$$

$$\begin{array}{rl} \log[E(Y_{t})] & =\alpha +\beta {\text{temp}}_{t,l}+\delta {\text{pol}}_{\text{cat},t}+\varepsilon {\text{temp}}_{t,l}\times {\text{pol}}_{\text{cat},t}\\ & \;+\text{ns}({\text{date}}_{t},7\times \text{year})+\zeta {\text{dow}}_{t}+\lambda {\text{pol}}_{01,t} \end{array}$$ (1)

where *E*(*Y_t_*) is the expected number of mortality at day *t*; *temp_t_*_,_*_l_* is a crossbasis function of temperature on day *t* with a lag period *l*; *pol_cat_*_,_*_t_* is an indicator variable representing a high or low level of the air pollutant (PM_2.5_ and O_3_) categorized based on the 75^th^ percentile cut-off; *date_t_* is time with 7 degrees of freedom (*dfs*) per year smoothed using a natural cubic spline *ns*; and *dow_t_* is the day of the week; *pol*_01,_*_t_* is the 2-day moving average of air pollutant concentrations. The *dfs* for date and temperature were selected based on the lowest QAIC value.

In the second stage, we pooled the first-stage results in each city using a random-effects meta-analysis to obtain an average association. We present the results as relative risks (RR) and 95% confidence intervals (CIs). To assess the effects of extreme temperatures, we calculated RR at the 1^st^ percentile of temperature (T01) relative to the 10^th^ percentile of temperature (T10) for extreme cold, and RR at the 99^th^ percentile of temperature (T99) relative to the 90^th^ percentile of temperature (T90) for extreme heat.

### Significance test for the presence and absence of effect modification

We examined whether there are significant differences between estimated values during high and low air pollutant concentration days. The following equation (Eq. 2) was used to calculate 95% CIs to test whether differences between estimated values for high/low concentration days are statistically significant followed by the presence or absence of effect modification [34, 35].

$$Q_{1}-Q_{2}\pm 1.96\sqrt{{{\mathrm{SE}}_{1}}^{2}+{{\mathrm{SE}}_{2}}^{2}}$$ (2)

Where *Q*_1_ and *Q*_2_ represent estimated values for the two categories (i.e., high-concentration and low-concentration days), and *SE*_1_ and *SE*_2_ are the standard deviations of each estimate. The p-values lower than the 5% alpha level are considered statistically significant.

We performed additional stratified analyses by sex and age. For age stratification, we used two categories: “early elderly” (those aged ≥65 and ≤74 years) and “late elderly” (those aged ≥75 years), given the increased susceptibility among the elderly population. We performed sensitivity analysis by using varying threshold values for defining the air pollution cut-off from the 50^th^ to the 90^th^ percentile [16, 17, 19]. We assessed the heterogeneity across the cities using the *I*^2^ test and Cochran Q test for heterogeneity [36]. We performed the analysis in R (version 4.0.0) using *dlnm*, and *mvmeta* packages [30, 33].

## Results

Table 1 shows the frequency and proportion of mortality by different causes, genders, and age groups from 2012–2018. The total number of deaths was 1,674,002, of which 25% were from cardiovascular diseases and 15.2% were from respiratory diseases. Slightly more than half of the cases (52.6%) were male. Most of the deaths (87.8%) occurred among the elderly population aged ≥65 years old, with 16.7% aged between 65 and 74 years, and 71.1% aged ≥75 years. A city-wise breakdown of the daily number and proportion of deaths is presented in Supplementary Table S1.

**Table 1:** Frequency (N) and proportion from total deaths (%) during 2012–2018.

**Outcome variables**	**Frequency (N)**	**Proportion (%)**
**Cause of mortality**		
All-cause mortality	1,674,002	100
Cardiovascular mortality	419,178	25.0
Respiratory mortality	254,343	15.2
**Gender**		
Male	880,293	52.6
Female	793,709	47.4
**Age group**		
Early elderly^a^	279,153	16.7
Late elderly^b^	1,191,052	71.1

^a^ Aged between 65–74 years old

^b^ Aged more than 75 years old

Table 2 shows the descriptive summary for temperature, PM_2.5_, and O_3_. The mean temperature across the cities was 16.1 °C, with a range from −1.5 to 32.1 °C. The mean PM_2.5_ and O_3_ was 13.9 µg/m^3^ and 41.2 ppb respectively, while the 75^th^ percentile of PM_2.5_ and O_3_ was 17.8 µg/m^3^ and 50.4 ppb, respectively. We used the latter as thresholds to define high or low levels of air pollution. Some cities in the Kanto region (i.e., Saitama, Chiba, Yokohama, Kawasaki, and Sagamihara) had very high levels of O_3_ (≥100 ppb) during the study period.

**Table 2:** Descriptive summary for temperature, PM_2.5_, and ozone during the study period 2012–2018.

**City**	**Temperature (°C)**					**PM_2.5_ (µg/m^3^)**					**O_3_ (ppb)**				
		
	**Mean (sd)**	**Min**	**P25**	**P75**	**Max**	**Mean (sd)**	**Min**	**P25**	**P75**	**Max**	**Mean (sd)**	**Min**	**P25**	**P75**	**Max**
Sapporo	9.5 (9.7)	−9.2	0.5	18.1	28	9 (6)	0	4.9	11.5	64.8	33.3 (10.3)	7.1	26.5	39.1	82.4
Sendai	13.2 (8.5)	−3.7	5.1	20.5	30.9	11.3 (7)	0	6.3	14.8	61	39.1 (11.7)	7	31.8	45.9	90.9
Saitama	15.7 (8.6)	−2.3	7.5	22.9	32.7	12.4 (7.2)	0	7.2	16.3	52.2	41.8 (17.4)	2.7	29.9	52	129.3
Chiba	16.6 (7.9)	0.3	9.3	22.9	32.1	11.9 (7.1)	0.7	6.7	15.4	50	40.4 (14.8)	3.7	30.5	49.8	104.7
Yokohama	16.6 (7.8)	0.3	9.3	22.9	32.2	15.3 (8.2)	0	9.9	19.3	59.6	38.9 (17.1)	1.9	26.7	49.8	137
Kawasaki	16.7 (7.7)	0.3	9.5	22.9	32.2	13.3 (5)	0	8.2	16.7	53.9	41.7 (17.2)	3.8	29.6	52.3	139.8
Sagamihara	16.7 (7.7)	0.3	9.5	22.9	32.2	12.1 (7.7)	0	6.8	15.9	59.6	40.5 (17.3)	4.4	28.1	51.4	126.2
Niigata	14.1 (8.7)	−2.8	5.9	21.9	33	10.5 (7.3)	0	5.5	13.2	86.6	44 (11.6)	12.7	36.7	49.7	101.2
Shizuoka	17.1 (7.5)	1.7	10.2	23.4	31.2	11.1 (6.7)	0.6	6.4	14	47.8	42.8 (14.2)	8.8	32.8	52.7	95.6
Hamamatsu	17 (7.8)	0	9.8	23.4	32.6	11.2 (6.9)	0	6.4	14.3	49.4	42.2 (14.2)	8.9	32.5	51.2	88.4
Nagoya	16.4 (8.6)	−1.1	8.2	23.7	33.3	14.3 (8.3)	0	8.4	18.5	69.3	42.1 (15.6)	1.8	31.2	52.6	97.5
Kyoto	16.4 (8.8)	−0.6	8.1	23.9	32.6	12.8 (7.2)	0.7	7.7	16.4	50.7	41.9 (15.3)	2.7	31.8	50.3	106.5
Osaka	17.1 (8.4)	0.1	9.2	24.2	32.7	15.8 (8.7)	0	9.3	20.6	64.2	39.6 (15.6)	2.8	28.7	49.6	107.1
Sakai	16.8 (8.4)	−0.2	9	23.9	32.5	17.6 (9.1)	1.6	11	22.2	73.9	40.6 (15.6)	3.1	29.5	50.4	100.6
Kobe	17.1 (8.3)	−0.8	9.4	24.1	32.5	13.5 (7.4)	0.1	8.1	17.3	57.5	41.8 (14.3)	6.6	31.8	50.5	96.7
Okayama	16.3 (8.6)	−2.2	8	23.6	32.3	15.7 (8.3)	0.5	9.6	20.1	56	41.5 (14.9)	3.6	31.6	50.2	94.6
Hiroshima	16.6 (8.5)	−2.2	8.7	23.8	32	15.5 (8.2)	1.9	9.5	19.9	66.1	43.2 (16.7)	2.4	31.9	53.6	98.9
Kitakyushu	16.8 (8)	−2.7	9.5	23.4	31.5	20.7 (9.9)	3.7	13.5	26	78.2	42.2 (14)	6.4	33.3	50.4	102.8
Fukuoka	17.5 (8)	−2	10.3	23.9	32.8	16.5 (9.1)	0.8	10	21	72.4	43.4 (14.6)	5.7	33.9	52.3	105.5
Kumamoto	17.3 (8.2)	−3	9.8	24.1	31.9	16.4 (9.2)	0.3	9.5	21.8	66	43.6 (15.4)	3.6	33.3	53.1	101.7

Overall	16.1 (8.5)	−9.2	8.5	23.1	33.3	13.9 (8.4)	0.0	7.8	18.0	86.6	41.2 (15.2)	1.8	30.9	50.3	139.8

**Fig. 2:**
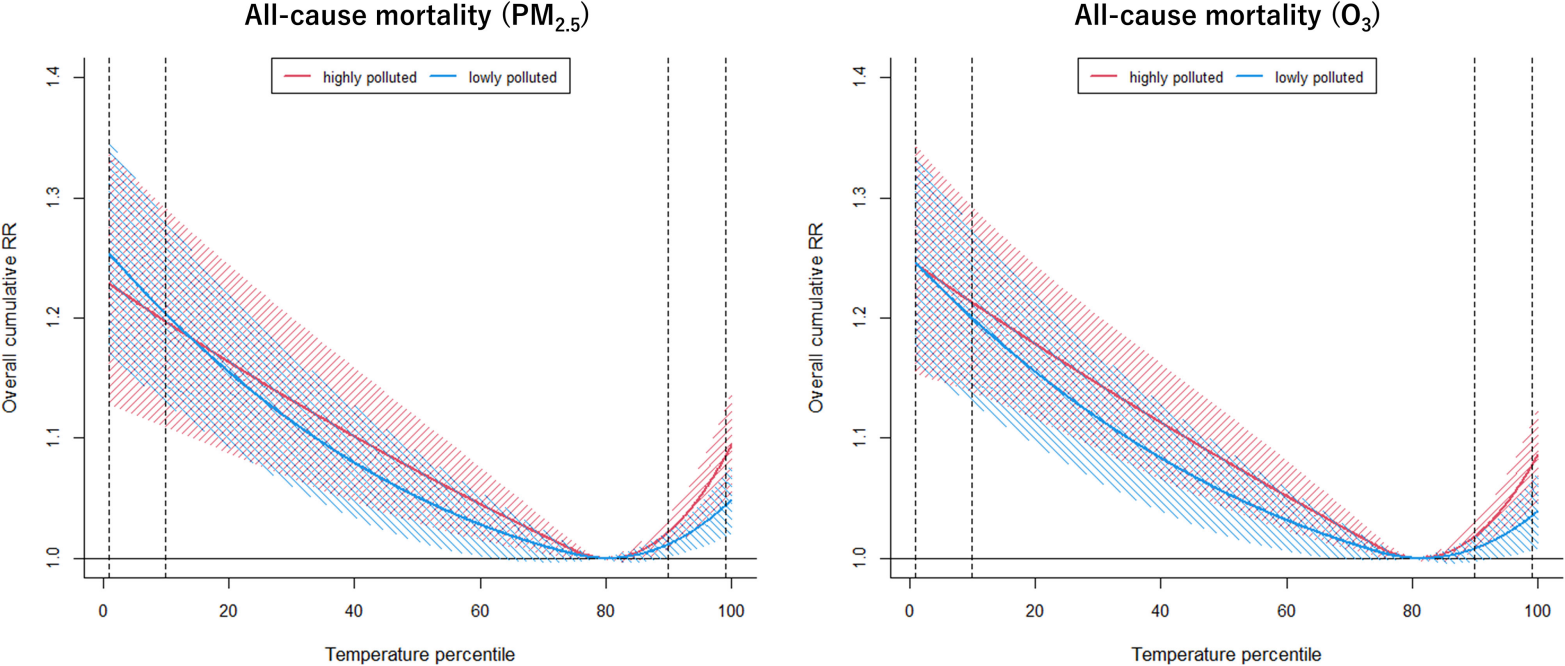
Overall pollution modified relative risk (RR) of temperature percentile and all-cause mortality. Left panel: effect modification from PM_2.5_; Right panel: effect modification from ozone; Blue color: days with low concentration; Red color: days with high concentration; Shaded region: 95% confidence interval; Dotted lines: 1^st^, 10^th^, 90^th^, and 99^th^ percentile of the temperature distribution.

Figure 2 shows the pooled results for all-cause mortality. We found a J-shaped association for temperature mortality with the lowest mortality risk approximately at the 80^th^ percentile of the temperature distribution and both extreme low and high temperatures increase the mortality risk. In general, we did not observe a significant modifying effect of air pollution on the association between temperature and mortality, as the temperature curves for high and low air pollutant levels mostly overlap for both PM_2.5_ and O_3_. The city-specific results of effect modification by PM_2.5_ and O_3_ are shown in Supplementary Fig. S1 & Fig. S2, respectively, while the overall temperature association without effect modification is shown in Supplementary Fig. S3.

Table 3 shows the pooled RRs and 95% CIs for extreme cold (T01 relative to T10) and heat (T99 relative to T90) by air pollutant level. Different cut-off percentiles ranging from the 50^th^ to the 90^th^ were used to classify low or high air pollution levels. When the 75^th^ percentile cut-off was used, the RRs for extreme cold were 1.03 (95% CI: 1.01, 1.04) and 1.04 (95% CI: 1.03, 1.06) on days with high and low PM_2.5_, respectively, but the difference is not statistically significant (p > 0.1). Similarly, for O_3_, the RRs for extreme cold are not statistically different between high and low O_3_ days. When different percentiles were used for the cut-off, we were not able to observe consistent evidence of effect modification by both air pollutants on extreme cold. On the other hand, PM_2.5_ and O_3_ may modify mortality risk associated with extreme heat, particularly O_3_, which showed strong differences between low and high pollutant levels at different percentile cut-off values (p < 0.05) (Table 3, Supplemental Fig. S4). Nonetheless, at 75^th^ percentile cut-off, the RRs for extreme heat are 1.06 (95% CI: 1.04, 1.09) and 1.03 (95% CI: 1.02, 1.05) on days with high and low PM_2.5_, respectively, with weak evidence of a difference (0.05 < p < 0.10). The RR for extreme heat appears slightly higher when O_3_ is high than when it is low (1.06 [95% CI: 1.03, 1.08] vs. 1.03 [95% CI: 1.01, 1.05]), despite weak evidence of a difference (0.05 < p < 0.10). As for the city-specific associations, we could only see a significant difference in the RR for extreme heat modified by PM_2.5_ for Sapporo city (0.75 [95% CI: 0.60, 0.95] vs. 0.97 [95% CI: 0.92, 1.02]), and Hiroshima city (1.17 [95% CI: 1.06, 1.29] vs. 1.03 [95% CI: 0.97, 1.11]). The city-specific results for effect modification on extreme cold and extreme heat can be found in Supplementary Table S2 and Table S3, respectively.

**Table 3:** Relative Risk (95% CI) of mortality attributable to extreme cold or extreme heat by strata of air pollutants representing low and high concentrations determined using different cut-off percentiles.

**Cut-off percentile**	**Extreme cold (1^st^ vs. 10^th^ percentile)**					

	**Effect modification by PM_2.5_**			**Effect modification by O_3_**		
	**Low concentration days**	**High concentration days**		**Low concentration days**	**High concentration days**	
P75	1.042 (1.025, 1.058)	1.026 (1.01, 1.043)		1.038 (1.022, 1.054)	1.026 (1.006, 1.048)	
P50	1.043 (1.026, 1.061)	1.032 (1.015, 1.048)		1.045 (1.03, 1.062)	1.025 (1.007, 1.043)	
P60	1.044 (1.027, 1.061)	1.027 (1.01, 1.045)		1.044 (1.027, 1.06)	1.016 (0.999, 1.033)	**
P70	1.043 (1.026, 1.06)	1.025 (1.009, 1.042)		1.04 (1.024, 1.056)	1.017 (0.998, 1.036)	*
P80	1.04 (1.024, 1.057)	1.028 (1.011, 1.045)		1.038 (1.022, 1.054)	1.029 (1.003, 1.055)	
P90	1.039 (1.023, 1.055)	1.029 (1.006, 1.051)		1.039 (1.023, 1.055)	1.019 (0.971, 1.069)	

**Cut-off percentile**	**Extreme heat (99^th^ vs. 90^th^ percentile)**					

	**Effect modification by PM_2.5_**			**Effect modification by O_3_**		
	**Low concentration days**	**High concentration days**		**Low concentration days**	**High concentration days**	

P75	1.032 (1.015, 1.050)	1.061 (1.035, 1.088)	*	1.027 (1.008, 1.046)	1.057 (1.034, 1.081)	*
P50	1.035 (1.013, 1.058)	1.047 (1.028, 1.067)		1.020 (1.000, 1.040)	1.062 (1.043, 1.081)	**
P60	1.029 (1.010, 1.049)	1.059 (1.038, 1.081)	*	1.019 (1.000, 1.038)	1.070 (1.050, 1.091)	**
P70	1.028 (1.011, 1.047)	1.067 (1.038, 1.096)	**	1.023 (1.004, 1.043)	1.068 (1.046, 1.091)	**
P80	1.035 (1.018, 1.053)	1.056 (1.025, 1.089)		1.031 (1.012, 1.051)	1.054 (1.028, 1.080)	
P90	1.036 (1.019, 1.054)	1.082 (1.033, 1.132)	*	1.031 (1.013, 1.051)	1.087 (1.046, 1.131)	**

* 0.05 < p < 0.1 for difference between strata of effect modifier

** p < 0.05 for difference between strata of effect modifier

For the effect of extreme cold, we could not see any effect modification by PM_2.5_ or O_3_ in any of the subgroups (Table 4). However, for the effect of extreme heat, we found a possible strong effect modification by O_3_ in the subgroup for cardiovascular mortality (p < 0.05), and a possible weak effect modification by PM_2.5_ and O_3_ among the females and elderly aged over 75 (0.05 < p < 0.1) (Table 5). Our test for heterogeneity showed that the results across the cities have low heterogeneity with an *I*^2^ value of <30% and a Cochran’s Q p-value of <0.05 (Supplemental Table S4).

**Table 4:** Relative Risk (95% CI) attributable to extreme cold by pollution levels and subgroup

**Extreme cold (1^st^ vs. 10^th^ percentile)**				

**Attributes**	**Effect modification by PM_2.5_**		**Effect modification by O_3_**	
	
	**Low concentration days**	**High concentration days**	**Low concentration days**	**High concentration days**
**Cause of mortality**				
Cardiovascular	1.09 (1.06, 1.12)	1.07 (1.04, 1.11)	1.08 (1.05, 1.11)	1.06 (1.02, 1.10)
Respiratory	1.01 (0.97, 1.04)	1.00 (0.96, 1.04)	1.00 (0.97, 1.04)	0.97 (0.92, 1.03)
**Gender**				
Male	1.05 (1.02, 1.07)	1.03 (1.01, 1.06)	1.04 (1.02, 1.07)	1.03 (1.00, 1.06)
Female	1.04 (1.02, 1.06)	1.02 (0.99, 1.04)	1.04 (1.01, 1.06)	1.02 (0.99, 1.05)
**Age group**				
65 to 74 years	1.06 (1.03, 1.09)	1.04 (1.00, 1.08)	1.06 (1.03, 1.09)	1.04 (0.99, 1.10)
≥75 years	1.04 (1.02, 1.06)	1.023 (1.00, 1.05)	1.04 (1.02, 1.06)	1.03 (1.00, 1.05)

**Table 5:** Relative Risk (95% CI) attributable to extreme heat by pollution levels and subgroup

**Extreme heat (99^th^ vs. 90^th^ percentile)**						

**Attributes**	**Effect modification by PM_2.5_**			**Effect modification by O_3_**		
	
	**Low concentration days**	**High concentration days**		**Low concentration days**	**High concentration days**	
**Cause of mortality**						
Cardiovascular	1.05 (1.02, 1.09)	1.05 (1.00, 1.11)		1.04 (1.01, 1.08)	1.11 (1.06, 1.17)	**
Respiratory	1.05 (1.00, 1.09)	1.06 (0.98, 1.14)		1.03 (0.99, 1.08)	1.09 (1.03, 1.15)	
**Gender**						
Male	1.04 (1.02, 1.06)	1.06 (1.03, 1.10)		1.04 (1.01, 1.06)	1.05 (1.02, 1.09)	
Female	1.02 (0.99, 1.05)	1.06 (1.03, 1.10)	*	1.02 (0.99, 1.05)	1.06 (1.03, 1.10)	*
**Age group**						
65 to 74 years	1.06 (1.02, 1.10)	1.07 (1.01, 1.13)		1.05 (1.01, 1.08)	1.07 (1.02, 1.13)	
≥75 years	1.03 (1.01, 1.05)	1.07 (1.04, 1.10)	*	1.03 (1.00, 1.05)	1.06 (1.03, 1.09)	*

* 0.05 < p < 0.1 for the difference between strata of effect modifier

** p < 0.05 for the difference between strata of effect modifier

## Discussion

In this study, we investigated the modifying role of PM_2.5_ and O_3_ on the short-term association between temperature and mortality in 20 urban areas of Japan. We found that the level of PM_2.5_ and O_3_ could modify mortality risks associated with extreme heat, but not extreme cold. The modifying effect of O_3_ on extreme heat was particularly apparent for mortality due to cardiovascular diseases, while females and the elderly populations might be more susceptible.

We found a difference in mortality risks between low and high levels of PM_2.5_ or O_3_ for extreme heat, but not for extreme cold. Previous studies also reported similar patterns in Germany, Europe, China, Australia, and Italy [16, 17, 19, 37, 38] where high temperatures were modified by PM_2.5_ and/or O_3_ where higher concentrations could increase the risk of mortality associated with high temperature. Our results agree with earlier studies that found a J-shaped pattern in how temperature and mortality risks are related in Japan and other countries, even after the effect modification of air pollution levels [16, 39–42]. The pooled results showed no significant difference of temperature effects between low and high PM_2.5_ concentration days. The results from the city-specific analysis suggested marginal variations in the effect modification of PM_2.5_ on extreme heat, and the effect modification was not statistically significant in most cities. The heterogeneity test suggested minimal variability among the 20 cities; however, further research covering more cities is necessary to understand the full extent of the disparities across Japan. City-specific information such as local industrial activity and mixture, traffic volume, and seasonal variations of the emissions of air pollutants could help shed light on the differences of effect modifications across cities. For instance, cities with higher industrial activities and traffic volume may experience different levels of air pollution. Additionally, seasonal variations in temperature and pollution levels could contribute to the differences in associations and interactions across cities.

We noticed some variation in how air pollutants modify the association between temperature and mortality, depending on the cause of death, sex, and age group. For cause-specific mortality, we found that O_3_ exhibited a stronger effect modification on cardiovascular mortality than PM_2.5_. This difference could be due to the different mechanistic reactions that temperature and air pollutants have on the circulatory and respiratory systems [17, 19, 43, 44]. We also found that females and the elderly population aged >75 groups may have a higher risk of death associated with extreme heat when exposure to extreme heat coincides with high levels of PM_2.5_ and O_3_. Possible explanations, beside the higher levels of exposure to air pollutants, include variations in susceptibility, adaptive capacity between males and females [39, 41, 43], and the higher frequency of chronic diseases, lower physiological reserve, and lower social support among the elderly [43, 45, 46].

Our results revealed that the effect modification by PM_2.5_ and O_3_ varied by the cut-off point used to define high or low levels of air pollutants. While we primarily used the 75^th^ percentile as the cut-off point, we conducted tests with other percentiles and found recurrent patterns. The shape of the temperature-mortality curves showed little variation across cut-off points for both PM_2.5_ and O_3_ (Supplementary Fig. S3). We observed strong evidence of PM_2.5_ effect modification on extreme heat at the 70^th^ percentile cut-off, while significant O_3_ effect modification on extreme heat was observed at the 50^th^, 60^th^, 70^th^, and 90^th^ percentiles. This implies that O_3_ exhibits a more consistent and stronger effect modification than PM_2.5_ on the temperature-mortality association. The choice of the cut-off point may depend on the distribution and variability of air pollution levels in different regions and seasons [19, 23]. A higher cut-off point may capture more extreme events, while a lower cut-off point may reflect more common exposures. Future studies might consider using multiple cut-off points or continuous measures of air pollution to examine the effect modification more comprehensively.

Our study is subject to numerous limitations. One limitation is that using fixed percentile cut-offs to classify air pollution levels may not reflect the non-linear association between air pollution and health effects. Future research could adopt spline functions or threshold models to allow for more flexible shapes of the exposure-response curves. However, it is worth noting that introducing an interaction term between the temperature cross basis and the air pollutant spline could add complexity to the analysis. Second, interpreting the modifying role of air pollutants on cold temperatures poses challenges. This difficulty arises from the prolonged delayed effects of cold and the comparatively short health impact of air pollutants. Careful consideration and perhaps innovative modeling approaches are needed to untangle the intricate interplay between cold temperatures and air pollutants. Third, our research covered only a subset of cities in Japan, and we did not consider other potential effect modifiers or confounders that might account for differences across cities. Future studies should consider extending the coverage to allow further assessment of factors such as socioeconomic status, population behaviors, healthcare, the mixture and activity of local industries, traffic volume and seasonal variations, which could have varying degrees of impact on the interactions between temperature and air pollutants. A longer timeframe may also provide insights into the changes in these interactive associations over time. These considerations could enhance future studies and provide more comprehensive insights into the research question.

## Conclusion

This study offers new perspectives on the short-term combined effects of temperature and major air pollutants on death rates in urban Japan. The results indicate that, while both extreme cold and heat increase the chance of death, high levels of PM_2.5_ or O_3_ may worsen the risk related to heat exposure, especially for mortality from cardiovascular diseases. The elderly and females are likely more susceptible. Further studies covering more areas, including those with low air pollution levels, are needed to confirm these results and understand the complex mechanisms behind how temperature and air pollution affect mortality.

## Supplementary information

**Additional file 1:** **Supplemental Figure S1.** First-stage prefecture-specific association between temperature percentile and relative risk of death considering effect modification of PM_2.5_ by city. **Supplemental Figure S2.** First-stage prefecture-specific association between temperature percentile and relative risk of death considering effect modification of Ozone by city. **Supplemental Figure S3.** Relationship between temperature percentile and relative risk of death without effect modification by city. **Supplemental Figure S4.** Relationship between temperature percentile and relative risk of death considering effect modification by PM_2.5_ (Panel A) and ozone (Panel B) by different percentile of pollutant to define the threshold. **Supplemental Table S1.** Descriptive results for daily number of deaths (% proportion from total deaths) in the 20 ordinance-designated Japanese cities during the study period 2012–2018. **Supplemental Table S2.** Relative risk of extreme cold (T01 vs. T10) by each city. **Supplemental Table S3.** Relative risk of extreme heat (T99 vs. T90) by each city. **Supplemental Table S4.** Heterogeneity tests across all 20 cities.

## Declarations

### Availability of data and material

The meteorological and air pollution data utilized for this study are publicly available from the Japan Meteorological Agency (https://www.data.jma.go.jp/stats/etrn/index.php) and the National Institute for Environmental Studies (https://www.nies.go.jp/igreen/) respectively. The mortality data is available from the corresponding author upon reasonable request. The data are not publicly available due to health information that could compromise the privacy of individuals.
