# Supplementary material for: The effect modification of PM2.5 and ozone on the short-term associations between temperature and mortality across the urban areas of Japan
Source: Environ Health Prev Med. 2024 Oct 26;29:57. doi: 10.1265/ehpm.24-00108 (PMC11524749; doi:10.1265/ehpm.24-00108)
Supplement: Supplementary file 1 — Additional file 1: Supplemental Figure S1. First-stage prefecture-specific association between temperature percentile and relative risk of death considering effect modification of PM2.5 by city. Supplemental Figure S2. First-stage prefecture-specific association between temperature percentile and relative risk of death considering effect modification of Ozone by city. Supplemental Figure S3. Relationship between temperature percentile and relative risk of death without effect modification by city. Supplemental Figure S4. Relationship between temperature percentile and relative risk of death considering effect modification by PM2.5 (Panel A) and ozone (Panel B) by different percentile of pollutant to define the threshold. Supplemental Table S1. Descriptive results for daily number of deaths (% proportion from total deaths) in the 20 ordinance-designated Japanese cities during the study period 2012–2018. Supplemental Table S2. Relative risk of extreme cold (T01 vs. T10) by each city. Supplemental Table S3. Relative risk of extreme heat (T99 vs. T90) by each city. Supplemental Table S4. Heterogeneity tests across all 20 cities. [file ehpm-29-057-s001.docx]

**Supplementary Material**

**Short-term association between temperature and mortality: effect modification by PM_2.5_ and Ozone in urban areas of Japan**


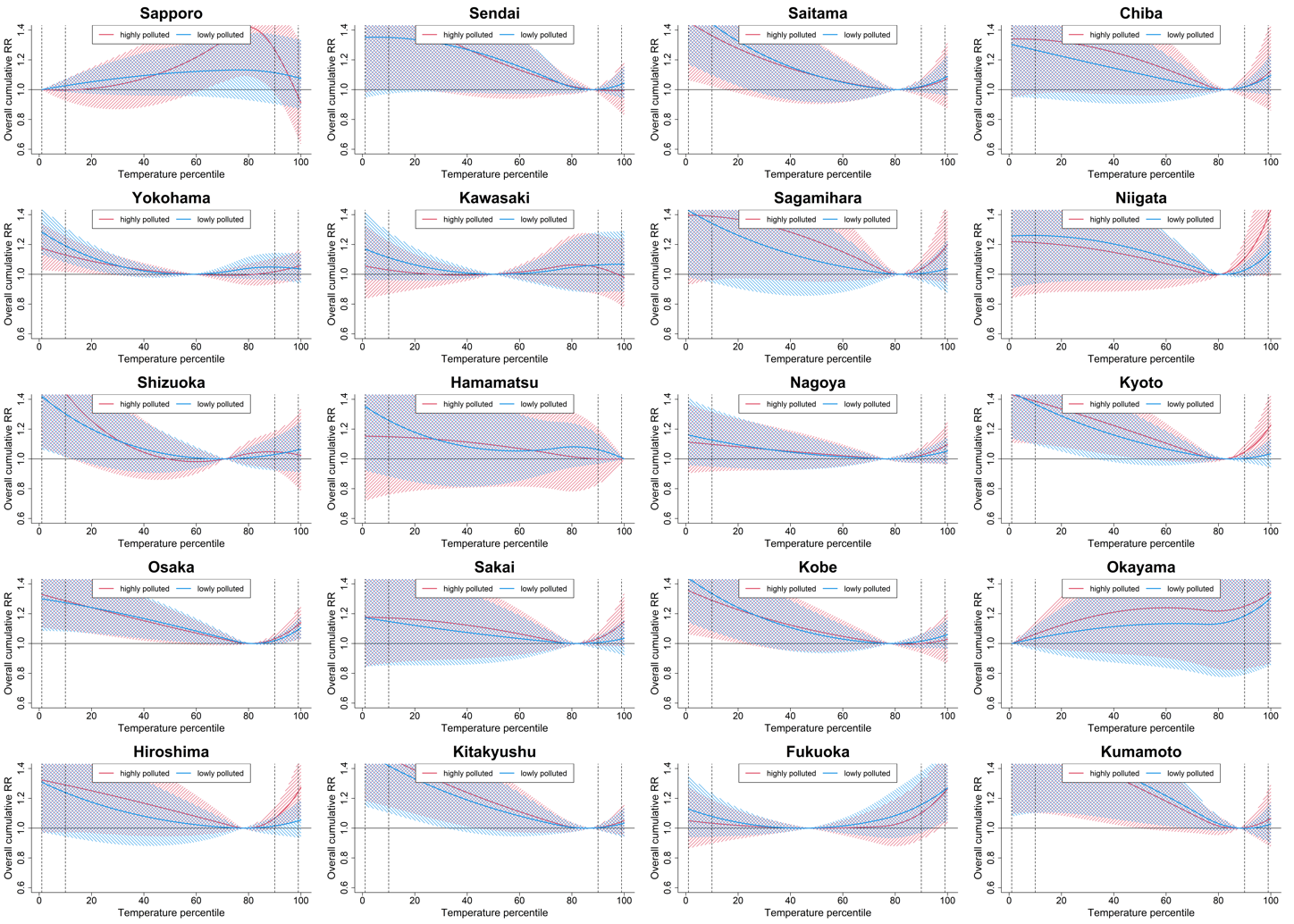


Supplemental Figure S1. First-stage prefecture-specific association between temperature percentile and relative risk of death considering effect modification of PM_2.5_ by city (red line during high PM_2.5_ concentration, blue line during low PM_2.5_ concentration, shaded area indicates 95% confidence interval for each)


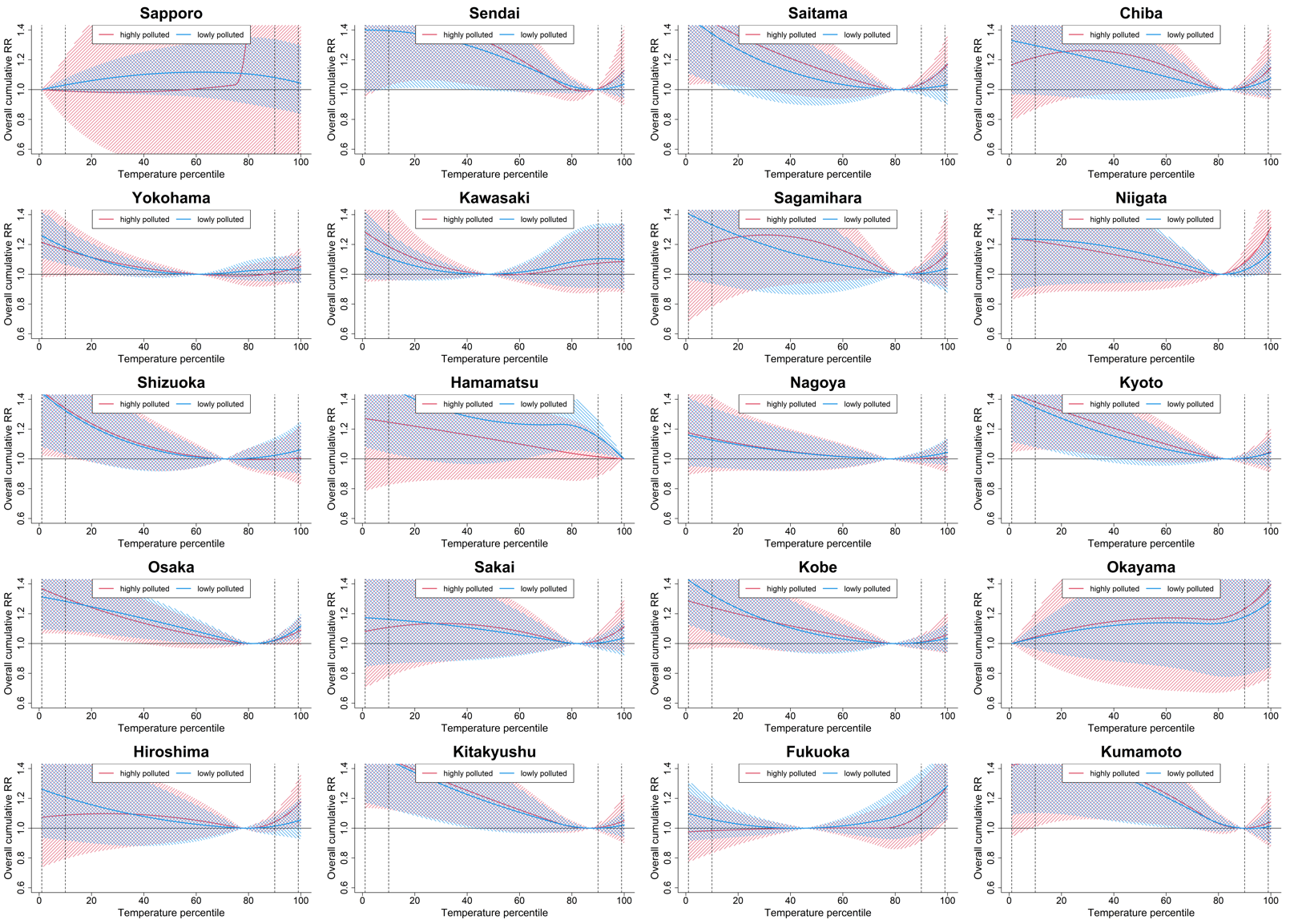


Supplemental Figure S2. Relationship between temperature percentile and relative risk of death considering effect modification by ozone by city (red line during high ozone concentration, blue line during low ozone concentration, shaded area indicates 95% confidence interval)


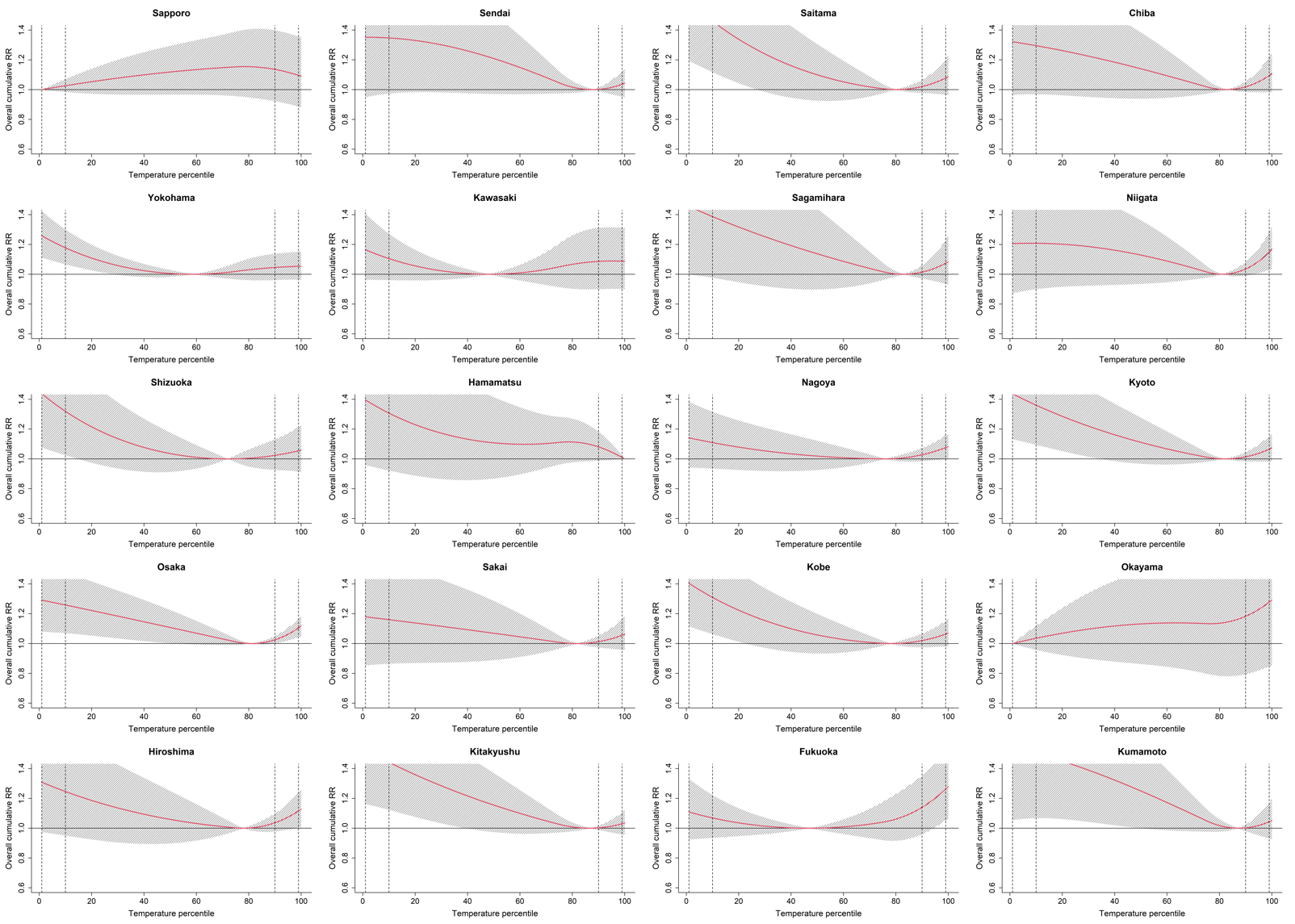


Supplemental Figure S3. Relationship between temperature percentile and relative risk of death without effect modification by city (red line indicates the central estimates, shaded area indicates 95% confidence interval)

| **A )** | 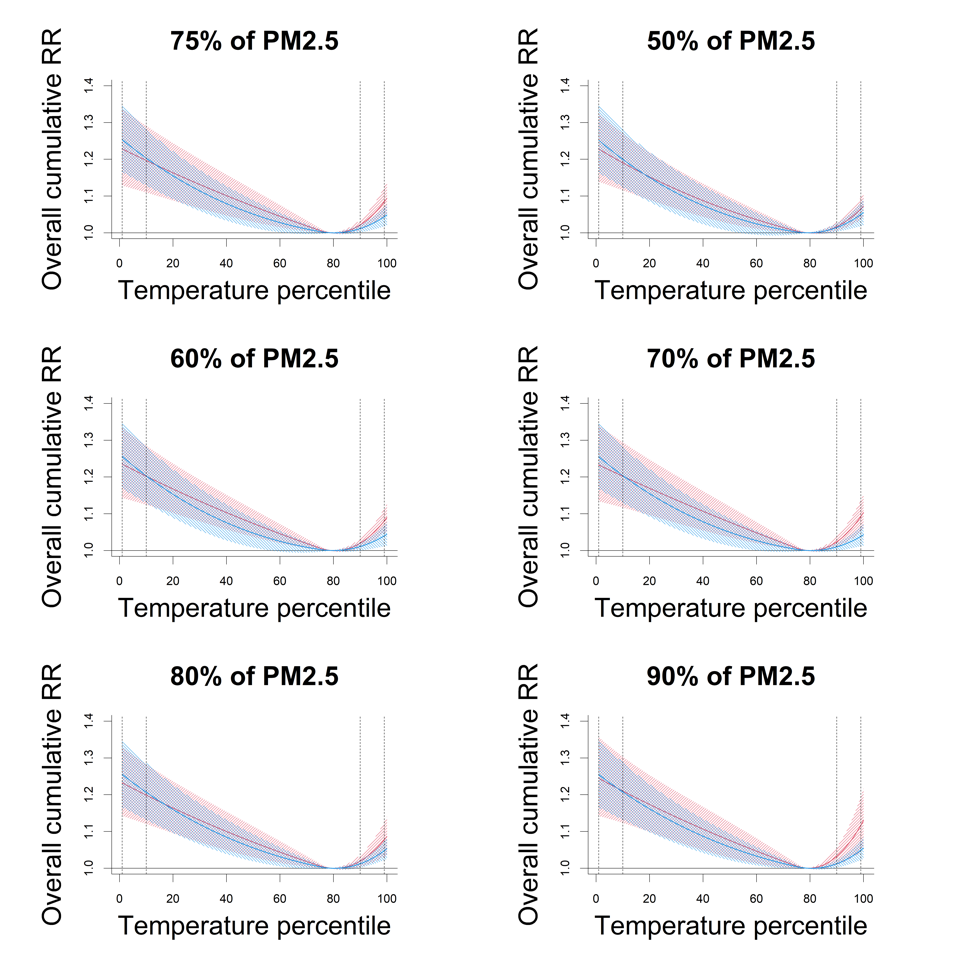 | **B )** | 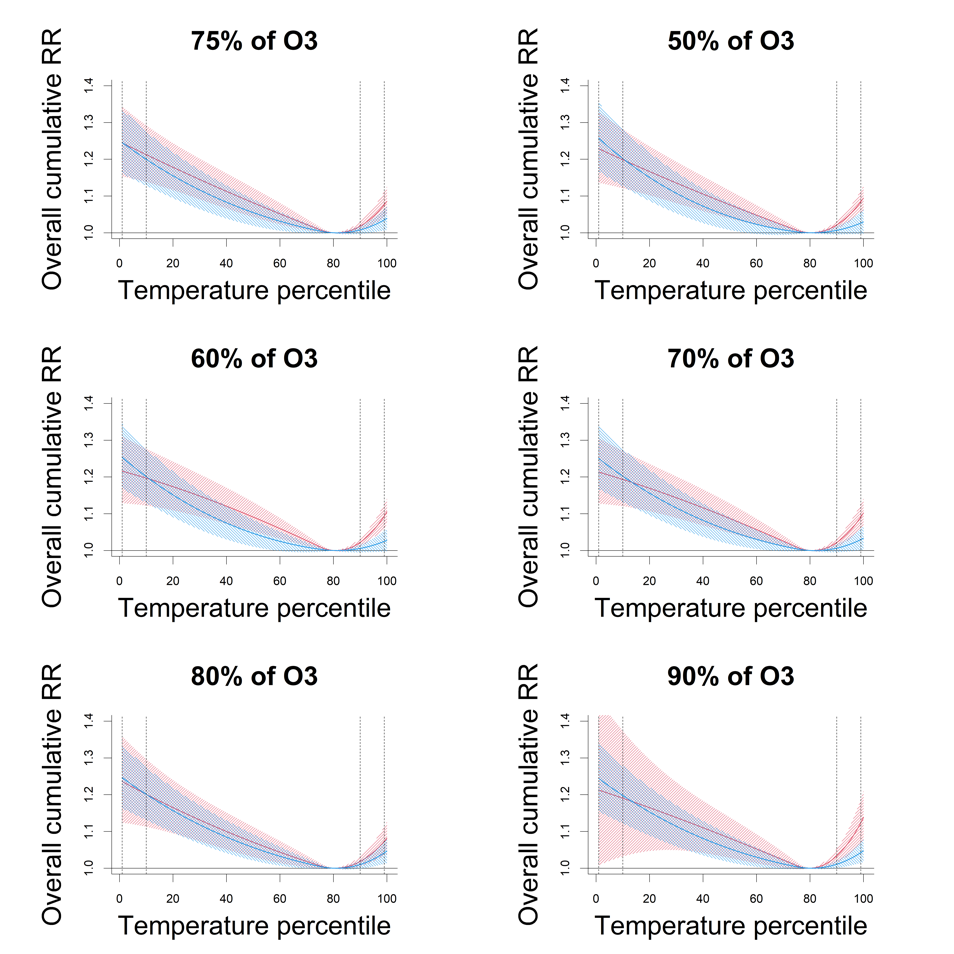 |
| --- | --- | --- | --- |

Supplemental Figure S4. Relationship between temperature percentile and relative risk of death considering effect modification by PM_2.5_ (Panel A) and ozone (Panel B) by different percentile of pollutant to define the threshold (red line during high pollutant concentration, blue line during low pollutant concentration, shaded area indicates 95% confidence interval). The dotted lines represent the 1^st^, 10^th^, 90^th^, and 99^th^ percentile of the temperature distribution.

**Supplemental Table S1.** Descriptive results for daily number of deaths (% proportion from total deaths) in the 20 ordinance-designated Japanese cities during the study period 2012-2018.

| **City** ^a^ | **All-cause** | **Cardiovascular** | **Respiratory** | **Male** | **Female** | **Early elderly^b^** | **Late elderly^c^** |
| --- | --- | --- | --- | --- | --- | --- | --- |
| Sapporo | 124780 | 31939 (25.6%) | 17798 (14.3%) | 64823 (51.9%) | 59957 (48.1%) | 20809 (16.7%) | 87228 (69.9%) |
| Sendai | 58975 | 15943 (27.0%) | 7261 (12.3%) | 30759 (52.2%) | 28216 (47.8%) | 8969 (15.2%) | 42035 (71.3%) |
| Saitama | 69677 | 17711 (25.4%) | 10647 (15.3%) | 37746 (54.2%) | 31931 (45.8%) | 12165 (17.5%) | 48289 (69.3%) |
| Chiba | 56431 | 15514 (27.5%) | 8448 (15.0%) | 31280 (55.4%) | 25151 (44.6%) | 10841 (19.2%) | 38026 (67.4%) |
| Yokohama | 215285 | 53416 (24.8%) | 30711 (14.3%) | 116210 (54.0%) | 99075 (46.0%) | 36227 (16.8%) | 152305 (70.7%) |
| Kawasaki | 63073 | 15831 (25.1%) | 9180 (14.6%) | 34803 (55.2%) | 28270 (44.8%) | 11022 (17.5%) | 43132 (68.4%) |
| Sagamihara | 34299 | 9286 (27.1%) | 5110 (14.9%) | 19078 (55.6%) | 15221 (44.4%) | 6491 (18.9%) | 23109 (67.4%) |
| Niigata | 50849 | 13472 (26.5%) | 6826 (13.4%) | 25760 (50.7%) | 25089 (49.3%) | 7177 (14.1%) | 38485 (75.7%) |
| Shizuoka | 53854 | 14387 (26.7%) | 7610 (14.1%) | 27847 (51.7%) | 26007 (48.3%) | 8186 (15.2%) | 40173 (74.6%) |
| Hamamatsu | 47618 | 12518 (26.3%) | 6477 (13.6%) | 24664 (51.8%) | 22954 (48.2%) | 6600 (13.9%) | 36348 (76.3%) |
| Nagoya | 146501 | 34413 (23.5%) | 21909 (15.0%) | 77476 (52.9%) | 69025 (47.1%) | 24987 (17.1%) | 104121 (71.1%) |
| Kyoto | 98477 | 26640 (27.1%) | 14465 (14.7%) | 49236 (50.0%) | 49241 (50.0%) | 15348 (15.6%) | 73034 (74.2%) |
| Osaka | 194550 | 47777 (24.6%) | 34037 (17.5%) | 106142 (54.6%) | 88408 (45.4%) | 36345 (18.7%) | 133046 (68.4%) |
| Sakai | 55893 | 14037 (25.1%) | 9684 (17.3%) | 29670 (53.1%) | 26223 (46.9%) | 10422 (18.6%) | 38841 (69.5%) |
| Kobe | 105639 | 25774 (24.4%) | 16052 (15.2%) | 54181 (51.3%) | 51458 (48.7%) | 16930 (16.0%) | 77009 (72.9%) |
| Okayama | 39343 | 10083 (25.6%) | 6345 (16.1%) | 19811 (50.4%) | 19532 (49.6%) | 5877 (14.9%) | 29499 (75.0%) |
| Hiroshima | 69917 | 18195 (26.0%) | 10583 (15.1%) | 35660 (51.0%) | 34257 (49.0%) | 11345 (16.2%) | 50251 (71.9%) |
| Kitakyushu | 75251 | 17154 (22.8%) | 12921 (17.2%) | 37923 (50.4%) | 37328 (49.6%) | 11625 (15.4%) | 55358 (73.6%) |
| Fukuoka | 79610 | 16574 (20.8%) | 12795 (16.1%) | 40594 (51.0%) | 39016 (49.0%) | 13175 (16.5%) | 54951 (69.0%) |
| Kumamoto | 33980 | 8514 (25.1%) | 5484 (16.1%) | 16630 (48.9%) | 17350 (51.1%) | 4612 (13.6%) | 25812 (76.0%) |
| Overall | 1674002 | 419178 (25.0%) | 254343 (15.2%) | 880293 (52.6%) | 793709 (47.4%) | 279153 (16.7%) | 1191052 (71.1%) |

^a^ From north to south

^b^ Early elderly: aged ≥ 65 and ≤ 74 years

^c^ Late elderly: aged ≥ 75

**Supplemental Table S2.** Relative risk of extreme cold (T01 vs. T10) by each city

| **City ^a^** | **Effect modification by PM_2.5_** | |  | **Effect modification by O_3_** | |  |
| --- | --- | --- | --- | --- | --- | --- |
|  | **Low concentration** | **High concentration** |  | **Low concentration** | **High concentration** |  |
| Sapporo | 0.97 (0.93, 1.02) | 1 (0.93, 1.08) |  | 0.97 (0.93, 1.01) | 1.01 (0.82, 1.25) |  |
| Sendai | 1 (0.93, 1.08) | 1.07 (0.94, 1.22) |  | 1 (0.93, 1.08) | 1 (0.88, 1.14) |  |
| Saitama | 1.09 (1.03, 1.16) | 1.07 (0.99, 1.15) |  | 1.09 (1.02, 1.15) | 1.06 (0.95, 1.18) |  |
| Chiba | 1.03 (0.96, 1.1) | 1 (0.92, 1.09) |  | 1.03 (0.96, 1.1) | 0.96 (0.87, 1.06) |  |
| Yokohama | 1.08 (1.04, 1.11) | 1.04 (1, 1.08) |  | 1.07 (1.03, 1.1) | 1.04 (0.98, 1.11) |  |
| Kawasaki | 1.05 (0.99, 1.11) | 1.03 (0.96, 1.1) |  | 1.06 (1, 1.12) | 1.08 (0.99, 1.18) |  |
| Sagamihara | 1.06 (0.98, 1.15) | 1 (0.91, 1.11) |  | 1.05 (0.98, 1.14) | 0.96 (0.83, 1.1) |  |
| Niigata | 1 (0.93, 1.07) | 1.01 (0.91, 1.11) |  | 1 (0.93, 1.07) | 1.02 (0.91, 1.13) |  |
| Shizuoka | 1.09 (1.02, 1.17) | 1.16 (1.03, 1.31) |  | 1.09 (1.02, 1.17) | 1.09 (0.98, 1.2) |  |
| Hamamatsu | 1.07 (1, 1.15) | 1 (0.9, 1.11) |  | 1.06 (0.99, 1.14) | 1.02 (0.91, 1.14) |  |
| Nagoya | 1.03 (0.99, 1.07) | 1.01 (0.97, 1.06) |  | 1.03 (0.99, 1.07) | 1.03 (0.96, 1.11) |  |
| Kyoto | 1.06 (1.01, 1.11) | 1.03 (0.97, 1.09) |  | 1.06 (1, 1.11) | 1.04 (0.95, 1.13) |  |
| Osaka | 1.02 (0.98, 1.06) | 1.03 (0.99, 1.07) |  | 1.02 (0.99, 1.06) | 1.05 (0.98, 1.12) |  |
| Sakai | 1.02 (0.95, 1.1) | 1 (0.94, 1.08) |  | 1.01 (0.94, 1.08) | 0.98 (0.87, 1.1) |  |
| Kobe | 1.08 (1.03, 1.13) | 1.05 (0.99, 1.11) |  | 1.08 (1.02, 1.13) | 1.03 (0.96, 1.12) |  |
| Okayama | 0.97 (0.89, 1.05) | 0.94 (0.86, 1.02) |  | 0.96 (0.89, 1.04) | 0.96 (0.83, 1.11) |  |
| Hiroshima | 1.06 (0.99, 1.12) | 1.03 (0.96, 1.1) |  | 1.04 (0.98, 1.11) | 0.99 (0.9, 1.09) |  |
| Kitakyushu | 1.06 (1, 1.13) | 1.06 (1, 1.12) |  | 1.06 (1, 1.12) | 1.05 (0.97, 1.13) |  |
| Fukuoka | 1.05 (0.99, 1.1) | 1.02 (0.96, 1.08) |  | 1.04 (0.98, 1.1) | 0.99 (0.92, 1.07) |  |
| Kumamoto | 1.02 (0.93, 1.11) | 1.03 (0.95, 1.13) |  | 1.03 (0.94, 1.12) | 0.98 (0.88, 1.09) |  |

* 0.05 < p < 0.1 statistically significant difference between effect modifiers

** p < 0.05 statistically significant difference between effect modifiers

^a^ From north to south

**Supplemental Table S3.** Relative risk of extreme heat (T99 vs. T90) by each city

| **City ^a^** | **Effect modification by PM_2.5_** | |  | **Effect modification by O_3_** | |  |
| --- | --- | --- | --- | --- | --- | --- |
|  | **Low concentration** | **High concentration** |  | **Low concentration** | **High concentration** |  |
| Sapporo | 0.97 (0.92, 1.02) | 0.75 (0.60, 0.95) | ** | 0.97 (0.92, 1.02) | NA^b^ |  |
| Sendai | 1.04 (0.96, 1.12) | 1.00 (0.86, 1.15) |  | 1.03 (0.95, 1.11) | 1.11 (0.92, 1.34) |  |
| Saitama | 1.06 (0.98, 1.15) | 1.05 (0.92, 1.20) |  | 1.02 (0.94, 1.12) | 1.11 (1.00, 1.23) |  |
| Chiba | 1.07 (0.98, 1.15) | 1.09 (0.91, 1.30) |  | 1.05 (0.97, 1.15) | 1.11 (0.96, 1.27) |  |
| Yokohama | 0.99 (0.95, 1.03) | 1.04 (0.99, 1.09) |  | 1.00 (0.96, 1.04) | 1.04 (0.98, 1.10) |  |
| Kawasaki | 1.01 (0.94, 1.09) | 0.94 (0.83, 1.07) |  | 1.00 (0.92, 1.08) | 1.01 (0.91, 1.12) |  |
| Sagamihara | 1.03 (0.92, 1.15) | 1.14 (0.97, 1.32) |  | 1.03 (0.92, 1.15) | 1.10 (0.95, 1.28) |  |
| Niigata | 1.11 (1.02, 1.20) | 1.25 (1.00, 1.58) |  | 1.10 (1.02, 1.19) | 1.19 (1.00, 1.41) |  |
| Shizuoka | 1.03 (0.96, 1.11) | 0.98 (0.83, 1.16) |  | 1.03 (0.95, 1.12) | 1.01 (0.90, 1.13) |  |
| Hamamatsu | 0.95 (0.88, 1.03) | 1.00 (0.85, 1.18) |  | 0.89 (0.81, 0.97) | 0.99 (0.87, 1.11) |  |
| Nagoya | 1.03 (0.98, 1.08) | 1.06 (0.98, 1.15) |  | 1.03 (0.98, 1.08) | 1.01 (0.94, 1.08) |  |
| Kyoto | 1.03 (0.97, 1.09) | 1.15 (1.02, 1.29) |  | 1.03 (0.97, 1.10) | 1.04 (0.95, 1.14) |  |
| Osaka | 1.07 (1.03, 1.12) | 1.09 (1.02, 1.16) |  | 1.08 (1.03, 1.13) | 1.06 (1.00, 1.13) |  |
| Sakai | 1.02 (0.95, 1.10) | 1.10 (0.99, 1.21) |  | 1.03 (0.96, 1.11) | 1.08 (0.97, 1.20) |  |
| Kobe | 1.04 (0.99, 1.09) | 1.02 (0.92, 1.14) |  | 1.02 (0.97, 1.08) | 1.04 (0.96, 1.13) |  |
| Okayama | 1.09 (0.99, 1.20) | 1.06 (0.94, 1.21) |  | 1.08 (0.97, 1.19) | 1.11 (0.98, 1.27) |  |
| Hiroshima | 1.03 (0.97, 1.11) | 1.17 (1.06, 1.29) | ** | 1.04 (0.96, 1.11) | 1.11 (1.02, 1.21) |  |
| Kitakyushu | 1.03 (0.96, 1.10) | 1.04 (0.96, 1.12) |  | 1.02 (0.96, 1.08) | 1.04 (0.93, 1.17) |  |
| Fukuoka | 1.09 (1.02, 1.16) | 1.13 (1.04, 1.23) |  | 1.10 (1.03, 1.17) | 1.15 (1.05, 1.27) |  |
| Kumamoto | 1.02 (0.93, 1.13) | 1.05 (0.90, 1.23) |  | 1.01 (0.92, 1.12) | 1.04 (0.89, 1.20) |  |

* 0.05 < p < 0.1 statistically significant difference between effect modifiers

** p < 0.05 statistically significant difference between effect modifiers

^a^ City ordered from north to south

^b^ Not estimated due to the low number of days

**Supplementary Table S4.** Heterogeneity tests across all 20 cities.

| **Effect modification** | ***I^2^*** | **Cochran-Q test (p-value)** |
| --- | --- | --- |
| PM_2.5_ | 27.2% | 0.0319 |
| O_3_ | 28.1% | 0.0274 |
